# Psychotherapy for Irritable Bowel Syndrome: A Systematic Review

**DOI:** 10.7759/cureus.51003

**Published:** 2023-12-23

**Authors:** Ethan Slouha, Bansari Patel, Ahmed Mohamed, Ziyad Razeq, Lucy A Clunes, Theofanis F Kollias

**Affiliations:** 1 Anatomical Sciences, St. George's University School of Medicine, St. George's, GRD; 2 Pharmacy, St. George's University School of Medicine, St. George's, GRD; 3 Pharmacology, St. George's University School of Medicine, St. George’s, GRD; 4 Medicine, St. George's University School of Medicine, St. George's, GRD; 5 Pharmacology, St. George's University School of Medicine, St. George's, GRD; 6 Microbiology, Immunology, and Pharmacology, St. George's University School of Medicine, St. George's, GRD

**Keywords:** hypnotherapy, mindfulness therapy, cognitive behavioral therapy, psychotherapy, irritable bowel syndrome

## Abstract

Psychotherapy has many forms, such as cognitive behavioral therapy (CBT), mindfulness therapy (MFT), and hypnotherapy, to name a few. Cognitive behavioral therapy is the gold standard in therapy-based treatment and is used for cognitive restructuring to reduce safety-seeking and avoidant behaviors. While the main application of psychotherapy is psychological disorders, recent studies have found that it is beneficial for somatic and physiological symptoms such as chronic pain or even irritable bowel syndrome (IBS). Irritable bowel syndrome is a common but debilitating gastrointestinal condition that has a prevalence of 12% in the United States and costs the average patient $9,776 annually in 2023. Irritatable bowel syndrome is a condition of exclusion but consists of abdominal discomfort or pain and must be associated with altered bowel habits as stated in the Rome IV criteria. At least half of these patients also exhibit extracolonic symptoms, most commonly psychological disorders like anxiety and stress. The true etiology of IBS is not understood, but ideas such as the brain-gut axis, stress response system, and gut microbiota have been evaluated. Treatment of IBS is extensive and heavily relies on the patient-physician interaction, but pharmacologic therapies have been employed and are sometimes unsuccessful. Irritable bowel syndrome impacts an individual as a whole, making them hesitate whether or not they eat a particular food or even go out to do an activity because of the unpredictable bowel pattern. Finding a better solution is essential to improving the patient's quality of life (QoL), especially by addressing how they perceive the illness, how they adjust to it, and even how they determine what foods to consume. This paper aims to evaluate whether or not psychotherapy can be employed to improve all aspects of IBS, as well as if it can reduce the cost of IBS treatment.

## Introduction and background

Psychotherapy

Psychotherapy is the intentional and informed application of interpersonal stances and clinical methods derived from the core psychological principles, offering treatment approaches that focus on cognitions, behaviors, relationships, emotions, and/or other personal characteristics [1]. Psychotherapy dates back to ancient Greece, and many cultures appreciate the tools employed by psychotherapy, but it became more developed in the 18th century [2]. The core of psychotherapy is the transformation of non-adaptive reasoning for an individual's problems into more adaptive and new ones [3]. It is considered the central and first-line treatment for most psychiatric conditions [1]. The main motive for going through psychotherapeutic treatment is to alter the general level of functions and reduce the symptoms of suffering while offering newly acquired clarity [3]. Psychotherapy has provided a way to describe personal experiences, which has led to the creation of fundamentally new ways of conceiving oneself [2]. Psychotherapy transforms experiences to enable coping and more favorable functioning while allowing individuals to be more adaptive [3]. However, the mechanisms by which psychotherapy creates change are still up for debate [3]. Still, psychotherapy is an effective intervention and the main approach in somatic and mental health care management [3]. There are multiple types of psychotherapy, but it has been proposed that factors such as patients’ expectations, understanding, trust, expertise, and the patient-therapist relationship explain their effectiveness [3].

Cognitive behavioral therapy (CBT) is one type of psychotherapy that dates back to 1960 and is coined as the gold standard therapeutic approach as it is effective in numerous psychiatric disorders such as anxiety, depression, eating disorders, and personality disorders [4]. Cognitive behavioral therapy was developed as psychologists noticed that patients with mental illness verbalize their thoughts, which come across as cognitive distortions [4]. Cognitive behavioral therapy is based on a common-sense model of relationships between cognition, emotion, and behavior, focusing on three aspects: automatic thoughts, cognitive distortions, and underlying beliefs of schemas [4]. Cognitive behavioral therapy sessions have personalized formats based on patients but, in general, start with a check on mood and a brief update, connecting previous sections, setting up a collaborative agenda, discussing homework assigned from previous sessions, and then diving into a conversation about problems experienced during the week and offering alternative ways to think and approach the problems [4]. One main function is to help eliminate safety-seeking and avoidant behaviors that typically prevent correcting faulty beliefs, reducing stress-related disorders, and improving mental health [5].

Mindfulness meditation goes back centuries within Buddhist practice. In the late 20th century, it was enhanced as a Western intervention to treat mental and even physical illnesses as mindfulness therapy (MFT) [6]. Mindfulness therapy was first applied to treat chronic pain by Kabat-Zinn. It was then extended into psychiatry to prevent depression relapse in association with cognitive modalities [6]. Mindfulness therapy is developed to train individuals to cultivate and incorporate mindfulness into their daily lives to appreciate and live in the moment [6]. Two components include attention regulation and openness to present experience, thus adopting the mentality of openness and acceptance towards the observed experience [6]. Mindfulness therapy has shown great success in the treatment of anxiety and depression [7]. Hypnotherapy is another form of psychotherapy involving hypnosis, an awake state of consciousness where a person’s attention is separate from their immediate environment and is absorbed by inner experiences such as cognition, imagery, and feelings [8]. It allows for a meditative state where individuals can learn to access their consciousness deliberately for therapeutic purposes [8]. This can lead to alleviating anxiety by accessing relaxation and calmness, helping cope with medication side effects, and even easing pain or other symptoms [8].

Irritable bowel syndrome

Irritable bowel syndrome (IBS) is the most commonly diagnosed gastrointestinal condition, with a prevalence of 12% in the United States and, as of 2023, costing individual patients an average of $9,776 [9, 10, 11]. Irritable bowel syndrome drastically reduces the patient’s quality of life (QoL) and can also negatively impact the patient's financial resources and society at large [11]. In 2010, IBS accounted for over 2 million clinic visits in the United States, consisting of emergency departments, primary care, and hospital outpatient departments [11]. Financial estimates from lost productivity, IBS management, and lost leisure time also amount to over one billion dollars [11]. Irritable bowel syndrome is a disease of exclusion consisting of abdominal discomfort or pain and is associated with altered bowel habits. Irritable bowel syndrome is diagnosed through the Rome IV criteria, which consists of three days a month within the last three months and is also associated with two or more of the following: onset associated with alteration in frequency of stool, improvement of abdominal discomfort or pain with defecation, and/or an onset accompanied by a change in the appearance or form of stool [10, 11]. Irritable bowel syndrome is classified into three categories: constipation, diarrhea, and mixed bowel pattern, but all present with bloating, symptoms brought on by food intake, distention, and a change in stool pattern and pain location over time [10]. In addition, extracolonic symptoms like psychological disorders like anxiety and depression affect 40%-60% of patients [11].

Irritable bowel syndrome is a multifactorial disorder, including inflammation, gastrointestinal dysmotility, altered intestinal microbiota, and visceral hypersensitivity [11]. The etiology is quite broad and not clearly understood, but visceral sensation, motility, psychosocial distress, and brain-gut interaction are thought to play a role in the development [10]. Due to stress being a significant contributor, the stress response system, which consists of the hypothalamic-pituitary-adrenal axis and autonomic nervous system, has been thoroughly investigated [11]. Another possible mechanism is altered gut immune activation and the colonic and intestinal microbiome, as individuals often report symptoms worsening upon eating. Foods leading to short-chain, highly fermentable carbohydrates that are poorly absorbed are associated with gastrointestinal symptoms present in patients with IBS [10]. The brain-gut axis has also been investigated as it consists of cross-talk between the central nervous system and the autonomic nervous system, such as the enteric nervous system, and patients usually have disturbances in autonomic and central functions, peptides, peripheral factors, and hormones [11].

Treatment of IBS needs to be individualized with a significant contribution towards patient education, reassurance for treatment, and management through a strong patient-clinician relationship [10, 11]. The goal of treatment is to resolve symptoms such as pain, cramping, bloating, and constipation or diarrhea, as there is no cure, but current pharmacological management typically provides suboptimal relief [10, 11]. Abdominal pain is usually prescribed antispasmodics, serotonin-selective reuptake inhibitors, peppermint oil, or tricyclic antidepressants [10, 11]. Constipation is usually treated with fiber supplements, chloride channel activators, guanylate cyclase C agonists, polyethylene glycol, and psyllium [10, 11]. Diarrhea is usually prescribed with opioid agonists, probiotics, antibiotics, mixed opioid agonists/antagonists, bile salt sequestrants, and 5-HT3 agonists [10, 11]. Antibiotics such as rifaximin can also be used, as they lead to less diarrhea and abdominal pain, supporting the proposed theory that bacterial overgrowth plays a role in IBS [10]. Non-pharmacological interventions, including psychotherapy, have become a dominant presence in the treatment of IBS to promote the mind-body connection, exercise, and diet modification [11].

Aim

Irritable bowel syndrome is a rather debilitating condition that accounts for a large number of emergency department and primary care physician visits in the United States. Despite these constant visits, minimal relief has been achieved as pharmacological management consistently proves to be suboptimal. Irritable bowel syndrome has also been associated with psychological comorbidities such as anxiety and depression that lead to further deterioration. Interestingly, psychotherapy is also quite successful at managing physiological conditions, as it alters the way the patient perceives and acts concerning their condition. Because of this ability, psychotherapy has been applied as an additional treatment for IBS, and there are numerous studies assessing the efficacy of various forms of psychotherapy. This paper aims to evaluate the success, efficacy, and cost-effectiveness of psychotherapy, specifically CBT, MFT, and hypnotherapy for IBS and IBS-associated symptoms, and to evaluate research that has compared the different psychotherapies.

## Review

Methods

The following systematic review was conducted with strict adherence to the Preferred Reporting Items for Systematic Reviews and Meta-Analyses (PRISMA) guidelines. This included a planned and thorough search of the current literature found in PubMed, ScienceDirect, and ProQuest between January 1, 2013, and November 1, 2023. The keywords for the search were ‘psychotherapy for IBS’, ‘cognitive behavior therapy for IBS’, ‘hypnotherapy for IBS’, and 'mindfulness therapy for IBS’. The investigation was centered on peer-reviewed observational and interventional publications. Publications not written in English, published before 2013, and duplicates were excluded. After the procurement of the publications, they were evaluated based on their title, abstract, study, and full-text availability. The preliminary inquiry into the databases used resulted in 27,763 publications. The abstracts of the publications were cross-referenced with the specific keywords selected, leading to specific publications that addressed the aim of this review. A total of 28 publications were collected according to the criteria stated below.

Inclusion Criteria

The publications were selected based on the following criteria: studies performed on humans, publications between 2013 and 2023 focused on psychotherapy outcomes for IBS, peer-reviewed observational or interventional studies, and full-text availability.

Exclusion Criteria

**Figure 1:**
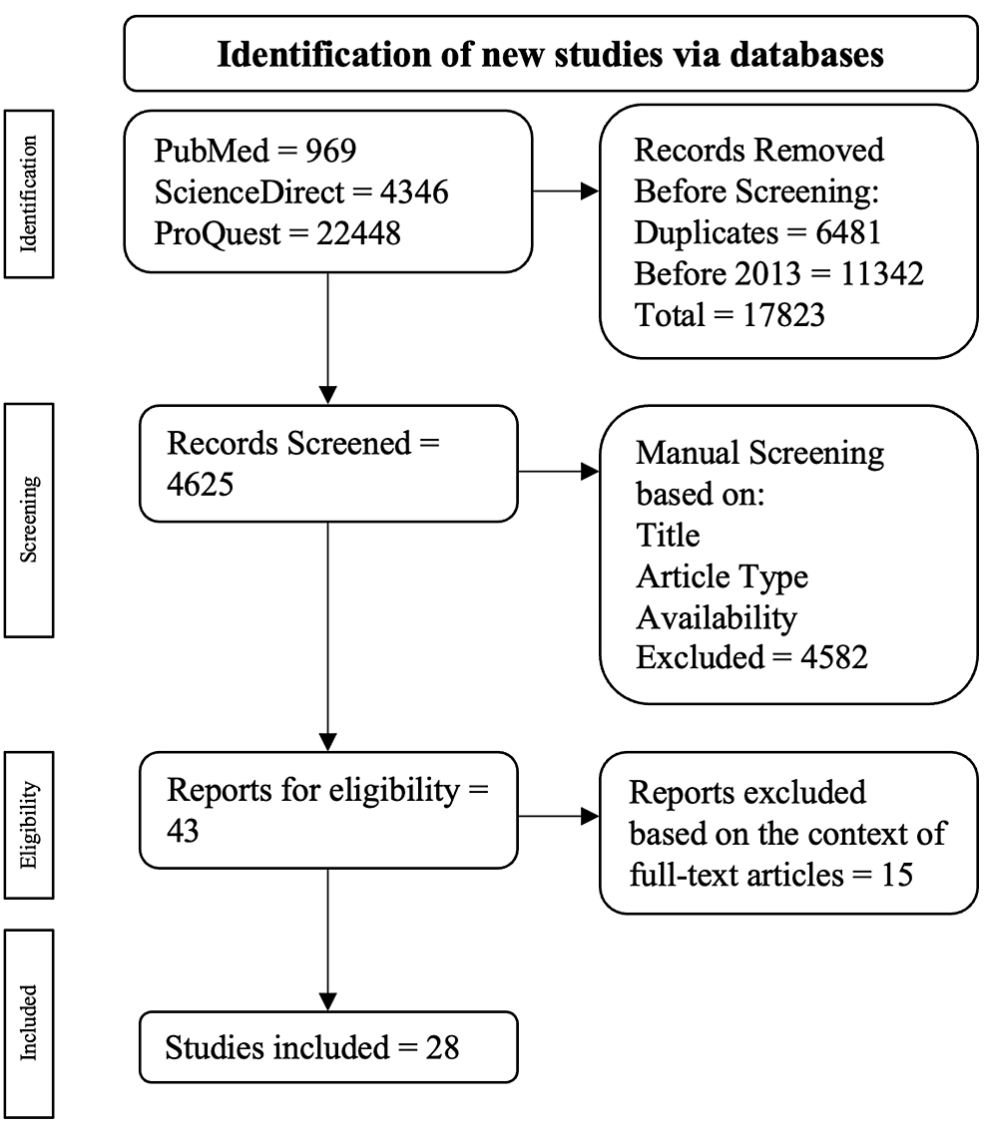
Algorithm employed using stated inclusion and exclusion criteria The flowchart was adapted as per PRISMA guidelines [12]. PRISMA: Preferred Reporting Items for Systematic Reviews and Meta-Analyses

The following criteria were used to exclude the publications: duplicates, non-English articles, and lack of full-text availability. The process of procurement using the inclusion and exclusion criteria is drawn out in Figure 1.

Bias

All publications acquired were assessed for bias through the Grading of Recommendation, Development, and Evaluation (GRADE) scale, and due to the small sample sizes of the majority of studies, a moderate bias rating was determined.

Results

A total of 27,763 articles were populated; 969 were from PubMed, 4,346 were from ScienceDirect, and 22,448 were from ProQuest. Among the exclusions, 6,481 were duplicate publications, and 11,342 were published before 2013. This resulted in 17,823 publications being excluded throughout the automatic screening procedure, leading to 4,625 publications for manual screening. Publications were then evaluated manually based on their title, study, and full-text accessibility, resulting in 43 publications being chosen for eligibility for full-text analysis. Ultimately, 28 publications were selected.

Cognitive behavioral therapy was found to be significantly successful at reducing the severity and frequency of IBS symptoms and also reducing debilitating pain. Cognitive behavioral therapy allowed for the modification and reduction of stress, depression, and anxiety in patients, allowing them to approach their diet with a clear mind, further reducing IBS symptoms. Dietary adjustments also alter the gut microbiota, which may contribute to IBS symptoms. Mindfulness therapy and hypnotherapy were also significantly successful at reducing the frequency and severity of IBS, but no studies were focused on the mechanisms responsible for their success. Few studies made comparisons between the different therapies. It was observed that MFT was superior to CBT and hypnotherapy when it came to long-term management and improvement in IBS symptoms. Hypnotherapy was found to be as successful as CBT; however, the effects of the therapy developed slowly. Mindfulness therapy was also found to be superior to dialectical behavioral therapy, emotional regulation, and positive psychotherapy. Compared to educational support, hypnotherapy was more successful in symptom control. However, one study that evaluated the blood oxygen level-dependent state following colonic distention found no significant differences. The articles analyzed for this review are presented in Table 1 with summaries of their findings and conclusions.

**Table 1:** Summary of articles used in this review per PRISMA Guidelines CBT: Cognitive behavioral therapy; IBS: irritable bowel syndromes; MFT: mindfulness therapy; PC: pain catastrophizing; QoL: quality of life; VSI: visceral sensitivity index; AA: Act awareness; ICBT: internet-based cognitive behavioral therapy; ICBT-WE: Internet-based cognitive behavioral therapy without exposure; QALY: quality-adjusted life year; MC-CBT: minimal contact cognitive behavioral therapy; S-CBT: standard cognitive behavioral therapy; ANS: autonomic nervous system; IBS-SSS: IBS symptom severity scale; CACE: complier average causal effect; PRISMA: Preferred Reporting Items for Systematic Reviews and Meta-Analyses [12]

	Author	Country	Design & Study Population	Findings	Conclusion
1	Boersma et al., 2016 [13]	USA	Experimental Study (n = 13)	Psychoeducation and MFT improve gastrointestinal symptoms, PC, and quality of life in IBS by 70%.	Psychoeducation and MFT improve gastrointestinal symptoms, PC, avoidance behavior, and quality of life.
2	Dehkordi et al., 2017 [14]	Iran	Experimental Study (n = 64)	There was significant improvement in QoL-IBS in the post-treatment period and follow-up stages and in severity and frequency of symptoms, but not in the follow-up stage in patients with CBT and drug therapy.	CBT, in addition to drug therapy, can be beneficial in improving the QoL, severity, and symptoms of IBS patients.
3	Henrich et al., 2020 [15]	UK	Experimental Study (n = 67)	The MFT group experienced notably larger decreases in IBS symptoms and enhancements in their QoL during the follow-up period compared to the waitlist group. The influence of the MFT's improvements in IBS symptoms was mediated by changes observed in VSI and PC after the treatment, coupled with the rise in non-judgmental awareness after the treatment.	The impact of mindfulness in reducing IBS symptoms could be due to its capacity to decrease unhelpful illness-related thought patterns and trigger alterations in how individuals perceive themselves by lessening tendencies towards biased interpretations of illness.
4	Hesser et al., 2017 [16]	UK	Randomized Control Trial (n = 309)	Among those assigned to the experimental condition, 55% were classified as compliers who completed the treatment as intended. The CACE analysis, which considered compliance status, revealed that the incremental effect of systematic exposure on IBS symptoms was greater than an intention-to-treat analysis that did not account for compliance.	The study findings suggest that the impact of systematic exposure on reducing IBS symptoms is substantial. To enhance overall treatment effectiveness in ICBT for IBS, focusing on individuals who prematurely discontinue treatment and tailoring interventions to improve their compliance may be beneficial.
5	Hunt et al., 2021 [17]	USA	Crossover Experiment (n = 121)	The immediate treatment group showed significant improvement compared to the waitlist control group in both gastrointestinal symptom severity and health-related QoL, depression, and stress.	Zemedy proves to be a successful approach for providing cognitive behavioral therapy to people with IBS, potentially enhancing the availability of this CBT treatment.
6	Kenwright et al., 2017 [18]	UK	Prospective Cohort Study (n = 104)	Patients who received CBT for bowel control anxiety had significant improvement with anxiety and IBS six months later. These patients also made improvements in their phobia scales.	Addressing bowel control anxiety associated with IBS through CBT not only improves anxiety but IBS symptoms as well.
7	Lee et al., 2019 [19]	Taiwan	Randomized Controlled Trial (n = 160)	ICBT and expressive writing showed a significant but small decrease in anxiety and depression at the end of the practicum and the three-month follow-up. Expressive writing showed a greater decrease in anxiety and depression in contrast to the ICBT at the end of the practicum. ICBT showed a greater improvement in reducing anxiety and depression at the three-month follow-up compared to the group for expressive writing.	There was a small decrease in anxiety and depression at the end of the practicum and three-month follow-up for the ICBT and expressive writing group compared to the waitlist control group.
8	Owusu et al., 2021 [20]	USA	Prospective Cohort Study (n = 25)	63.6% of subjects had a significant improvement overall. At two months of follow-up, cognitions and gastrointestinal-specific anxiety, along with depression and anxiety at three months, greatly improved.	ICBT significantly improved symptom severity, cognition, gastrointestinal-specific anxiety, depression, and anxiety.
9	Bonnert et al., 2017 [21]	Sweden	Randomized Control Trial (n = 101)	The analyses revealed a significant and noteworthy improvement from pretreatment to post-treatment in IBS-SSS for the ICBT group. After a follow-up period of six months, the results remained stable or showed further significant improvement.	ICBT utilizing exposure exercises is an effective approach for improving gastrointestinal symptoms and enhancing the quality of life in adolescents with IBS.
10	Jang et al., 2014 [22]	South Korea	Experimental Study (n = 76)	The experimental group receiving CBT had a more pronounced reduction in symptom frequency, distress, and daily life disability compared to the control group. There were no significant differences in dysfunctional attitude scores between groups.	CBT is a beneficial intervention for female patients with irritable bowel syndrome, as evidenced by reductions in symptom frequency, distress, and daily life disability.
11	Edebol-Carlman et al., 2018 [23]	Sweden	Experimental Study (n = 18)	CBT did not significantly impact ANS activity during induced visceral pain and cognitive stress. Sympathetic activity remained high, consistent with IBS characteristics, during both stressors. However, there was a significant decrease in levels of state and trait anxiety following the intervention. There were no significant changes in self-reported current or perceived stress.	In conclusion, the study suggests that face-to-face CBT for IBS primarily improved anxiety rather than affecting the autonomic stress response to experimentally induced visceral pain and cognitive stress.
12	Roger et al., 2023 [24]	USA	Clinical Trial (n = 436)	CBT reduced PC during treatment and significantly correlated with improvements in IBS outcomes.	Decreases in PC are linked to a reduction in the severity of IBS symptoms, as well as enhancements in overall symptom relief and improvements in the QoL specifically related to IBS.
13	Radziwon et al., 2022 [25]	USA	Randomized Control Trial (n = 358)	Completing homework for CBT was significantly associated with improvement of IBS symptoms and patient satisfaction, but it did not predict an early response to treatment.	Homework completion for CBT in treating IBS is crucial for the success of psychotherapy.
14	Jacobs et al., 2021 [26]	USA	Experimental study (n = 84)	There was increased fecal serotonin and *Clostridiales* in CBT responders and decreased *Bacteroides* compared to non-responders. Patients who responded to CBT had reduced functional connectivity in sensorimotor, brainstem, salience, and default mode networks.	CBT actually influences the brain-gut microbiome in IBS patients.
15	Naliboff et al., 2020 [27]	USA	Experimental Study (n = 55)	The study on mindfulness using the Five Factor Mindfulness Questionnaire showed a significant positive improvement in three out of five facets, especially the strongest predictor of QoL gastrointestinal symptoms in the AA facet for gastrointestinal symptoms and QoL. The gastrointestinal responder rate was 71%.	Regression analyses show that increasing present-moment focus and acting with awareness are the most important outcomes that help patients use mindfulness.
16	Zernicke et al., 2013 [28]	Canada	Experimental Study (n = 90)	There was a 30.7% reduction in the severity of IBS symptoms observed immediately post-MFT relative to the control.	Patients in MFT had a reduction in symptom severity that went from being constantly present to only being present occasionally.
17	Zomorrodi et al., 2015 [29]	Iran	Experimental Study (n = 24)	MFT is accompanied by greater self-confidence, optimism, life satisfaction, success, and improved coping skills and QoL.	MFT has a significant, positive effect on IBS patients’ long-term QoL.
18	Flik et al., 2018 [30]	Netherlands	Randomized Control Trial (n = 342)	Hypnotherapy demonstrated significant effectiveness compared to the control group at the three-month and 12-month assessments. In the per-protocol analysis, group hypnotherapy was as effective as individual hypnotherapy, meeting the criteria for non-inferiority.	This study implies that hypnotherapy should be regarded as a viable treatment option for patients with IBS, both in primary and secondary healthcare settings. Group therapy, in particular, has the potential to offer an effective and cost-efficient approach to treating IBS in a larger patient population.
19	Lovdahl et al., 2022 [31]	Sweden	Randomized Clinical Trial (n = 119)	Improvements in symptom severity, extracolonic symptoms, psychological symptoms, and QoL improved in both group and individual hydrotherapy performed by a nurse. Subjects were divided between individual and group hypnotherapy to compare effectiveness.	Nurse-led gut-directed hypnotherapy showed group hypnotherapy can be an effective alternative.
20	Mohebbi et al., 2021 [32]	Iran	Randomized Control Trial (n = 100)	The gastrointestinal symptom severity of the hypnotherapy group improved significantly between six and 15 weeks post-hypnotherapy. After 15 weeks of hypnotherapy, they also had an improvement in QoL.	Using hypnotherapy and medicinal treatment could be effective for health systems and patients.
21	Gerson et al., 2013 [33]	USA	Experimental Study (n = 75)	The study found a significant reduction in IBS symptoms at each assessment point. The researchers discovered that the initial severity score and Quality of Relationship Inventory Conflict (QRIC) score were directly correlated with a positive response to hypnotherapy.	Group hypnotherapy is an effective treatment for patients with IBS, as it leads to significant symptom reduction.
22	Zomorrodi et al., 2014 [34]	Iran	Quasi-Experimental Study (n = 36)	MFT was seen to be more effective at improving QoL and reducing symptoms of IBS as compared with CBT.	Compared to CBT, MFT demonstrated long-term improvements in the clinical symptoms of IBS as well as increased QoL.
23	Ghandi et al., 2018 [35]	Iran	Experimental Study (n = 24)	MFT stress reduction led to an improvement in the QoL-IBS and a reduction in the severity of their condition. The analysis showed that the difference in IBS outcomes between the MFT and control groups was statistically significant at the follow-up stage.	The study suggests that MFT stress reduction can be considered a novel, effective, and enduring psychotherapeutic approach to treating IBS.
24	Mohamadi et al., 2019 [36]	Iran	Randomized Control Trial (n = 76)	Based on the perceived stress scale and QoL-IBS, they showed significant differences. Perceived stress was significantly decreased for MFT compared to other groups, and great effects on quality of life showed higher scores in positive psychotherapy.	MFT and positive psychotherapy were more effective in decreasing stress and improving QoL.
25	Lowen et al., 2013 [37]	Sweden	Experimental Study (n = 64)	Symptom reduction success in blood oxygen level-dependent attenuation in the dorsal and ventral anterior insula during high-intensity distension occurred in both groups for hypnotherapy and education interventions. Hypnotherapy responders showed a greater blood oxygen level-dependent increase in the posterior insula, whereas educational responders had it in the prefrontal cortex pre-and post-treatment. Post-treatment, healthy controls had a similar response to distension.	Psychological interventions like hypnotherapy and education could help normalize the effect of processing abnormal visceral signals in irritable bowel syndrome and be mediated by different brain processes.
26	Dunlap et al., 2021 [38]	USA	Experimental Study (n = 436)	There was a significant decrease in the cost of $296 per patient in those treated with MC-CBT compared to S-CBT and a significant decrease in the cost of $109 per patient in those treated with MC-CBT compared to education/support. MC-CBT has already led to a better average in patient outcomes immediately and six months post-treatment.	MC-CBT might be the new wave of treatment for IBS through greater outcomes and decreased cost.
27	Sampaio et al., 2018 [39]	Sweden	Experimental Study (n = 101)	Providing ICBT to adolescents with IBS leads to enhancements in health-related QoL and results in minor gains in QALYs at a greater cost compared to waitlist control.	Given the robust evidence of its efficacy, the slight QALY improvements, and its cost-effectiveness, ICBT is likely to represent a valuable and cost-effective treatment option for this population.
28	Wallen et al., 2021 [40]	Sweden	Randomized Control Trial (n = 309)	ICBT cost 20% more than ICBT-WE at the six-month endpoint. For every point improved on the GSRS-IBS version of ICBT, costs or society would be reduced by $300. Cost-effectiveness had an 84% probability of occurring.	Incorporating exposure within CBT for IBS proves to be economically advantageous despite the potential requirement of additional therapist and patient engagement in the immediate term.

Discussion

Cognitive Behavioral Therapy

Several studies have solely evaluated the impact of CBT on IBS, with primary outcomes ranging from the Gastrointestinal Symptoms Rating Scale (GSRS), the IBS-Symptom Severity Score (IBS-SSS), symptom frequency, the Behavioral Response Questionnaire (BRQ), the Pain Catastrophizing Scale (PCS), QoL, depression, and anxiety. Cognitive behavioral therapy significantly improved GSRS and IBS-SSS, which include somatic symptoms like diarrhea, bloating, and constipation, with one study reporting that 69.2% of subjects showed >70% improvement from baseline, which was in range for other studies [13-20]. Cognitive behavioral therapy done specifically for anxiety with bowel control anxiety techniques also had a significant improvement in IBS symptoms [18]. Lee et al. observed that significant improvements were observed until 18 weeks into their study, and both expressive writing and internet-delivered CBT showed significant improvements in symptoms [19]. Dehkordi et al. observed that while there was initial improvement, this was not noticed at follow-up [14]. One study also evaluated additional factors that significantly attributed to the reduction in symptoms and found that elderly and female patients, employed patients, and patients with university education displayed a faster decline in symptoms [16]. There was also a significant reduction in the frequency of symptoms in patients following CBT, but Dehkordi et al. observed that this was not maintained at follow-up [14, 21, 22].

Other factors in patients with IBS were also assessed as they contribute to the disability, including the Stress Symptoms Rating Questionnaire (SSRQ) and PCS. Only one study found that following CBT, the SSRQ, which evaluates the stress caused by these symptoms, was not significantly lower than the baseline within the group [23]. The PCS was significantly reduced through treatment with CBT, with one study reporting that 76.9% of subjects had a greater than 70% reduction, indicating a decrease in pain associated with IBS [13, 15, 24]. The decrease in PSC was also correlated with global symptom improvement and improvement in QoL [24]. Quality of life is also a critical component of IBS that should be evaluated, as IBS can be debilitating. It was observed that CBT significantly improved QoL in patients with IBS, and this continued through to follow-up in all studies that assessed it [13-15, 17, 21, 22, 24].

Since IBS is debilitating, it takes a toll on the mental health of the individual. Addressing comorbid mental health problems can also improve the symptoms of IBS. About 61.5% of subjects showed a greater than 70% reduction in the BRQ from baseline, suggesting a successful decrease in avoidance behavior [13]. With CBT, there was a significant change at two months concerning gastrointestinal-specific anxiety, gastrointestinal-focused cognitions, and safety behaviors decreased except for unhelpful IBS avoidance behaviors; however, there was no clinical significance [20]. Depressive and anxiety symptoms improved significantly according to improvements in the Patient Health Questionnaire-9 (PHQ-9), Trait Anxiety Inventory Score (TAIS), State Anxiety Inventory Scores (SAIS), and the Depression Anxiety Stress Scale (DASS) in most studies [15, 17-21, 23]. Hunt et al., however, observed that the DASS anxiety subscale did not show significant improvement following CBT [17]. Jang et al. observed that with CBT, there was a significant decrease in dysfunctional attitude scores, which indicated an overall reduction in dysfunctional attitudes toward their IBS [22]. There was also an improvement in the amount of stress concerning their IBS, with a significant improvement in the Perceived Stress Scale (PSS) scores [21, 23].

Secondary outcomes of some studies included GI-specific catastrophizing, visceral anxiety, and fear of food, which were also significantly improved in patients receiving CBT [17]. These mediators also had significantly indirect effects on the QoL of patients [17]. Radziwon et al. observed that completion of CBT homework was not a predictor of early response to treatment but was associated with increased satisfaction with treatment at the conclusion [25]. Completed homework was also associated with higher levels of IBS symptom improvement, and this didn’t vary when comparing face-to-face and internet-based CBT [25]. One study reported that 4.4% of their subjects experienced a deterioration of symptoms at two months and remained unchanged at three months [20].

Cognitive behavioral therapy has been shown to cause changes in how an individual interacts, as patients undergoing CBT were more likely to have a lower carbohydrate intake, eat more fiber, and consume more total and monounsaturated fat, which aids in reducing the symptoms as their intestines are more likely to handle these dietary changes [26]. Cognitive behavioral therapy has also been found to alter the gut microbiota by increasing *Roseburia*, *Lachnobacterium*, and unclassified *Lachnospiraceae *while decreasing *Bacteroides*, *Parabacteroides*, and *Prevotella* [26]. These changes may contribute to greater brain connectivity between emotional regulation and central autonomic networks [26]. The exact mechanism of why CBT worked for the actual symptoms of IBS, including constipation, bloating, and diarrhea, is not clear nor mentioned in the studies. However, CBT is about rewiring how you think, so it could contribute to two possible mechanisms. First, the induction of different hormones resulting from decreased anxiety or depression further influences the gut through the brain-gut axis. Secondly, it alters how the patient thinks regarding what food they pick and consume, which also indirectly influences the symptoms they could experience.

Mindfulness

Another long-term technique is teaching patients IBS mindfulness concerning their actions and thought processes. Mindfulness therapy significantly improved IBS symptoms and IBS-SSS compared to control groups by up to 30.7% and was shown to be clinically meaningful [27, 28]. Zernicke et al. observed, however, that patients receiving mindfulness training had a reduction in symptom severity that went from being constantly present to only being present occasionally [28]. However, the statistical significance was not maintained at six months [28]. Participants who meditated at home had a positive change in IBS symptom severity [27]. Naliboff et al. also found that the subjects' expectations of the study outcome were positively associated with the IBS-SSS [27]. Additional scales consisted of the Nonjudge scale, Act Aware, and Observed, all showing a significant increase in pre- to post-treatment and post-treatment and follow-up correlating with the success of MFT [27]. Naliboff et al. also found that PCS and widespread somatic symptoms significantly decreased, with Act Aware being the strongest predictor for catastrophizing pain [27]. It was also observed that the greatest predictor of change in somatic symptoms was sex [27].

A tremendous improvement in the QoL of participants was also noted immediately post-treatment and at follow-up [27, 29]. The mindfulness techniques taught led to improved coping skills, which contributed to the improvement in QoL and the sustainability and lifelong effect [29]. Act Aware was also positively associated with the change in QoL, but only post-treatment (following eight weeks) and not in the follow-up [27]. Mid-treatment, IBS-SSS change was positively associated with a post-treatment change in IBS-QoL and Visceral Sensitive Index (VSI) [27]. Mindfulness techniques also alter psychiatric symptoms such as anger, depression, and anxiety [27, 29]. However, Naliboff et al. did not observe a significant change in depression; the Nonjudge scale was the greatest predictor of depressive symptoms [27]. They also found no significant change from post-treatment to follow-up and no significant effect on sex interaction [27]. The Act Aware scale was also the greatest predictor of the VSI, assessing fear and anxiety in the IBS [27]. The Nonjudge scale was specifically the strongest predictor for anxiety alone [27]. Like with depression, no significant effects of sex interaction or time were noted [27]. Mindfulness therapies are accompanied by greater self-confidence, optimism, life satisfaction, and success [29]. And like CBT, explanations as to why MFT works on somatic symptoms are unclear, and no mechanism has been proposed. Still, it may also influence patients' dietary choices, despite studies not evaluating this possibility.

Hypnotherapy

Hypnotherapy has also been used in an attempt to alleviate the symptoms of IBS in patients and has shown great responses. Between 33.3% and 71% of subjects qualified as responders to hypnotherapy, having adequate relief from most symptoms [30, 31]. In traditional one-on-one hypnotherapy, there was a significant difference in global symptoms only six weeks after the start of treatment [32]. Concerning symptoms and IBS-SSS, there was a significant improvement in the severity of symptoms in up to 50% of participants, with the greatest improvement being seen at the three-month mark [30-33]. Gerson et al. observed that a correlation analysis revealed that the attributing symptoms to psychological causes were inversely correlated with IBS-SSS, meaning that those who emphasized emotional influences had a lower IBS-SSS [33]. Notably, the reduction in various components of the IBS-SSS, including abdominal pain, distension, bowel habit, and interference in life experience, showed significant improvement [33]. In terms of predictors of treatment outcomes, the initial IBS-SSS was highly correlated with a reduction in IBS-SSS at one year, suggesting that patients with more severe IBS were more responsive to hypnotherapy [33]. When assessing the Mind-Body scale, results showed an inverse correlation between psychological attributions and IBS-SSS reduction, indicating that patients who attributed their symptoms to psychological factors were less likely to respond positively to hypnotherapy [33]. Physical factor attributions did not significantly correlate with the outcome of treatment [33].

The QoL significantly improved compared to the baseline for hypnotherapy participants [30, 31]. The severity of psychiatric symptoms was also significantly improved from baseline [31]. Psychiatric symptoms such as depression and anxiety specifically improved following this [30, 31]. Post-treatment, there was an improvement in gastrointestinal-specific anxiety [31]. At the three- and 12-month mark, patients reported fewer IBS-related work absences, fewer work hindrances, and better work efficiency than baseline compared to the control group [30].

A couple of studies compared the different formats of hypnotherapy, specifically individual and group. At visit five, 71% of subjects in individual hypnotherapy and 60% of group hypnotherapy participants were classified as responders, achieving adequate relief [31]. Another study found that only 40.8% of individual hypnotherapy participants and 33.2% of group hypnotherapy participants were responders [30]. In both groups, it was found that symptom severity improved slowly over time, but there was no significant difference between the groups [30, 31]. The symptom severity and frequency of abdominal pain, dissatisfaction with bowel habits, interference of life from bowel symptoms, and severity of bloating showed no difference between both groups [30, 31]. Both groups also showed improvement. The QoL, however, specifically physical functioning, did not improve with group hypnotherapy [31]. Improvement in depression was slightly greater in individuals who underwent hypnotherapy, and both led to significant improvement in anxiety [30, 31]. Participants in both groups showed improvements in IBS-related cognitions and self-efficacy compared with baseline, which can lead to better choices regarding daily activity and dietary choices, reducing somatic symptoms [30].

Comparing Therapeutic Techniques

Two studies compared CBT to other therapeutic techniques, such as MFT and hypnotherapy. It was found that while all three significantly improved symptom severity, MFT demonstrated long-term improvements in clinical symptoms [31, 34]. Compared to CBT, hypnotherapy slowly improved symptom severity over treatment, but no significant difference was observed [31]. All three showed significant improvement in QOL; however, like with symptom severity, mindfulness therapy displayed long-term improvements compared to CBT, and no difference was observed between CBT and hypnotherapy [31, 34].

Mindfulness therapy was also compared to emotional regulation, dialectic behavior therapy, and positive psychotherapy. Post-test IBS-SSS was significantly increased in MFT, dialectic behavioral therapy, and positive psychotherapy but was not reproducible in emotional regulation [35, 36]. This was correlated with a significant difference in IBS severity between MFT and emotional regulation [35]. Mindfulness therapy, dialectic behavioral therapy, and positive psychotherapy significantly improved the QoL; however, MFT only showed a significant improvement [35, 36]. Perceived stress and QoL scored lower in positive psychotherapy than in MFT and dialectic behavioral therapy [36]. The emotional regulation group did not significantly improve QoL at the post-test stage, possibly because QoL for IBS patients is influenced by various factors, including social relationships, job satisfaction, and mental health [35].

Only one study compared hypnotherapy with another therapeutic approach, education. Both hypnotherapy and the education group showed decreased symptom severity with no significant difference [37]. Both groups reported a significant decrease in gastrointestinal-specific anxiety, symptom intensity, and unpleasantness after procedures [37]. At the neurological level, pre- to post-symptom improvement in the VSI and a blood oxygen level dependent increase in the anterior insula had significant correlations between the improvement of gastrointestinal symptoms and a blood oxygen level dependent decrease in the hippocampus during high-intensity distension [37]. Blood oxygen level dependent signals during the expectation of distension and high-intensity distension were significantly reduced post-treatment in the ventrolateral and dorsolateral prefrontal cortex, ventral and dorsal anterior insula, amygdala, posterior insula, and hippocampus for all treatment responders [37]. However, the blood oxygen level-dependent was not increased pre- to post-treatment during the rectal distension [37]. There was significantly more blood oxygen level-dependent response for treatment responders than healthy control during high-intensity distention in the anterior mid-cingulate cortex, pregenual cingulate cortex, subgenual anterior cingulate cortex, and ventrolateral prefrontal cortex [37]. Thirteen hypnotherapy responders had a great blood oxygen level-dependent attenuation in the ventral and dorsal anterior insula pre- and post-treatment during the high-intensity distension [37]. In contrast, education responders saw a decrease in the ventral and dorsal anterior insula and a reduction in the ventrolateral prefrontal cortex [37]. Post-treatment differences, however, were not reported except for the ventrolateral prefrontal cortex [37].

Cost

An additional factor to consider with the addition of psychotherapy to IBS treatment is cost-effectiveness. Three studies evaluated this, but they took it a step further and compared internet-based CBT, standard CBT, and internet-based CBT without exposure. Internet-based CBT significantly improved overall symptoms compared to patients undergoing standard CBT and internet-based CBT without exposure [38-40]. This was also associated with enhancements in health-related QoL and resulted in minor gains in quality-adjusted life years [39]. Also, internet-based CBT yielded significant reductions in expenses at follow-up in two studies [38]. Sampaio et al. observed that the average cost of internet-based CBT per participant was $170.24 higher than that of the waitlist group but less than other treatments [39]. Comparing it to internet-based CBT with exposure, Wallen et al. found that for every $1 invested in internet-based CBT over internet-based CBT without exposure, the return amounted to $5.64 six months following treatment [40]. Given the robust evidence of its efficacy previously described, slight quality-adjusted life-year improvements, and its cost-effectiveness, internet-based CBT will likely present a valuable and cost-effective treatment option for IBS patients [39].

A limitation of this study was the lack of studies comparing the different therapeutic techniques to truly visualize the gradient of success between them. There was also an unequal number of studies, with the majority focusing on CBT, which hindered the power of mindfulness and hypnotherapeutic strategies concerning this study. There are numerous approaches to solving problems, but it’s about finding the best approach and strengthening its mechanism to improve the outcome. While there was some comparison, there was a lack of strength in highlighting one over the other, leading to the need to carefully interpret the results. It’s important to note that numerous other studies highlight new techniques, but not enough on each approach to add to the study, but may be more successful than the therapies included in this study.

## Conclusions

Psychotherapy is an established approach for many psychological conditions, consisting of techniques such as CBT, MFT, and hypnotherapy, to name a few. Irritatable bowel syndrome is a chronic and debilitating disease for many individuals across the globe and requires long-term care that escalates in intensity as they get older. An alternative approach to treatment has been proposed, implemented, and improved over the last 10 years, consisting of a psychotherapeutic approach towards controlling IBS symptoms and the associated psychiatric symptoms involved. Cognitive behavior therapy, MFT, and hypnotherapy have been shown to significantly improve symptom severity, QoL, PCS, and behavioral responses toward IBS. Cognitive behavioral therapy has the additional benefit of altering an individual's interaction with food intake, leading to alterations in the gut microbiota and dually improving IBS. Mindfulness therapy, though, has been shown to be superior to emotional regulation, dialectic behavioral therapy, and positive psychotherapy. One study also observed the neurological impact of hypnotherapy via evaluating blood oxygen during intestinal distention and compared this to educational-based therapy. They found no difference in blood oxygen level-dependent signals between groups except for the ventrolateral prefrontal cortex. Psychotherapy, specifically CBT, has also been shown to be a cost-effective treatment, significantly decreasing the amount of money needed to invest in treatment compared to standard treatment.

It's important to note that several other therapeutic techniques are being applied to treat IBS, but not enough research has been done regarding each individually to add to this paper. Although a lot more research needs to be done, especially revolving around the exact mechanisms as to why psychotherapy has provided such relief, it is clear from this review that the QoL of patients is significantly improved with no reported adverse effects. The pharmaceutical costs of multiple medications alone could debilitate the patient, but even then, pharmaceuticals may not provide the necessary relief, leading to refractory IBS. It has been shown that psychotherapy may also prove to be effective for refractory IBS, but more research is needed.
